# Supplementary material for: Generation of Conditional Knockout Alleles for PRUNE-1
Source: Cells. 2023 Feb 6;12(4):524. doi: 10.3390/cells12040524 (PMC9954577; doi:10.3390/cells12040524)
Supplement: Supplementary file 1 [file cells-12-00524-s001.zip › cells-2197788 supplementary materials.pdf]

Legends for Supplemental Figures

Figure S1. The sequence of donor DNAs used for generating conditional mouse allele of the *Prune1*. The sequence of homologous arms and *lox*P site are indicated. The red front represents the sequence used for gRNA synthesis, and the green front indicates the protospacer adjacent motif (PAM).

Figure S2. Genotyping of targeted alleles through zygote electroporation. Zygote electroporation of 100 ng/µL of gRNA, 200 ng/µL of donor DNA and 50 ng/µL Cas9 in a 10 µL volume of Opti-MEM® was used for both targeting. The ratio of electroporated zygotes developed to the blastocytes in both targeting was around 50%. gRNA/donor DNA used for targeting 5' *lox*P showed significantly higher efficiency on generating homozygous knock-in (indicated by red arrows) than the one used for targeting 3' *lox*P.

Figure S3. The DNA sequencing data to confirm the corrected insertion of *lox*P sites into the *Prune1* locus in the $Prune1^{F/F}$ allele.

Figure S4. Generation of the $Prune1^{\Delta exon6}$ allele. (a) The $Prune1^{F/F}$ allele was bred with *EIIa-Cre* mice for the excision of exon 6, resulting in the $Prune1^{\Delta exon6}$ allele. (b) PCR genotyping for the detection of the excision of exon 6 in the $Prune1^{\Delta exon6}$ allele. Lane 1, the wild-type with a ~1.4 kb PCR product; Lane 2, the $Prune1^{\Delta exon6/\Delta exon6}$ homozygote in which a single 329 bp PCR product was detected.

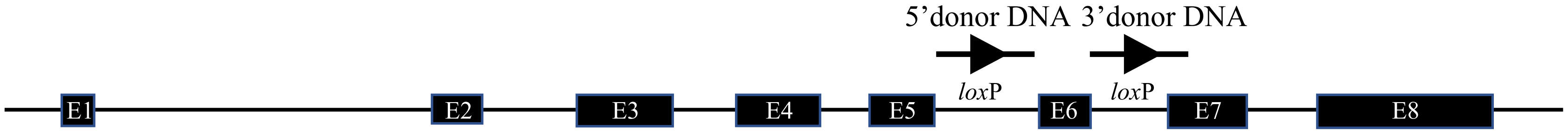

5’ donor DNA

cacacccagtaaagctgctcttcttttaccctcctacaaagtccctcaatc**ATAACTTCGTATAGCATACATTATACGAAGTTAT**

Homologous arm — *lox*P

tacaggcagccatttaactattgtgacctgattgttagcattttagagttttctttat

Homologous arm

3’ donor DNA

tgctcatggcacaaggggattggtttgtggtttgttagtagtcgtgctcaa**ATAACTTCGTATAGCATACATTATACGAAGTTAT**

Homologous arm — *lox*P

agaaggaagaaattagattcagaaatctctctctcccttctcccctctccttct

Homologous arm

Figure S1

(a) Zygote electroporation with gRNA/Donor for 5’ *lox*P site

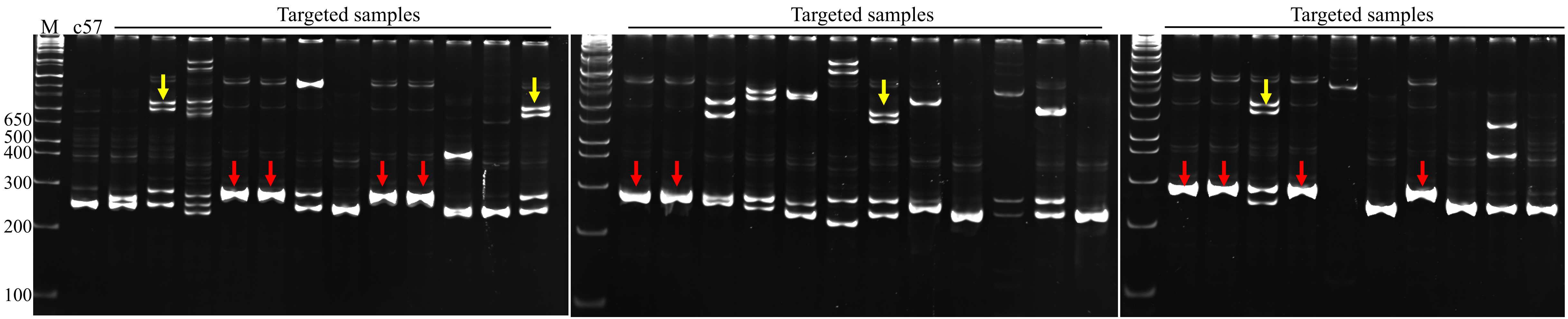

10/34 samples showed homozygous knock-in (indicated by red arrows)
4/34 samples showed heterozygous knock-in (yellow arrows)
20/34 samples contained Indel or other mutations

(b) Zygote electroporation with gRNA/Donor for 3’ *lox*P site

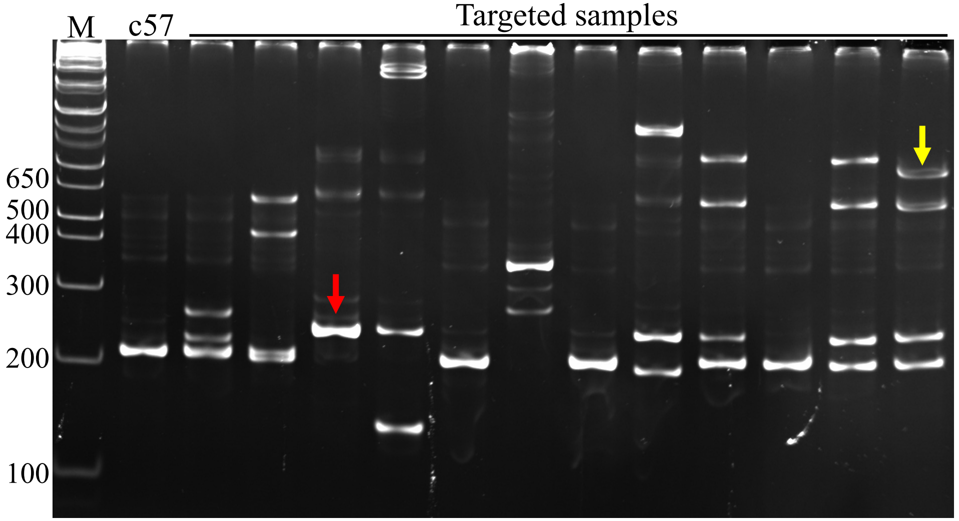

1/12 samples showed homozygous knock-in (indicated by red arrow)
1/12 showed heterozygous knock-in (yellow arrow)

Figure S2

Quality Graph

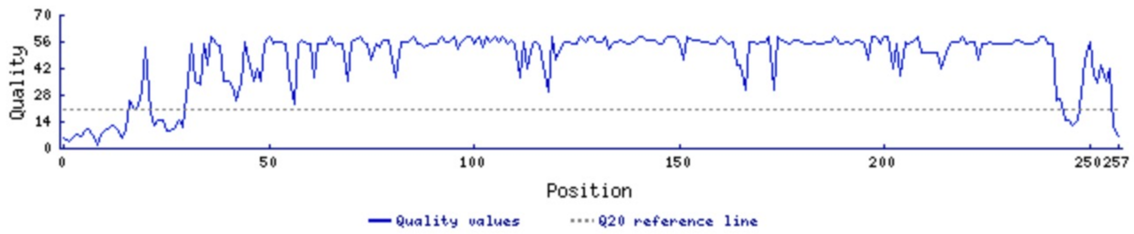

Read Length	258 b	Trimmed Length	243 b	Pos. 16-258
Q20	227 b	Q20/len	0.88	
Q40	197 b	Q40/len	0.76	

Sequence

Previous Revision	n/a	Current Revision	1	Next Revision	n/a	Latest Revision	1

Links: View Revision History

```
>5P_1RR_WPG1372-user_added CHROMAT_ID=1601340
gcatgggckatctcactcctgagctcaccagcctcttcctgcagagcactgggctcaagg     60
gtgtgcaccacccccacacccagtaaagctgctcttcttttaccctcctacaaagtccct    120
caatcataacttcgtatagcatacattatacgaagttattacaggcagccatttaactat    180
tgtgacctgattgttagcattttagagttttctttattattggatatgtgtgggtgttt     240
tgcctgatgtatgtcaaa
```

Targeted 5' loxP

Quality Graph

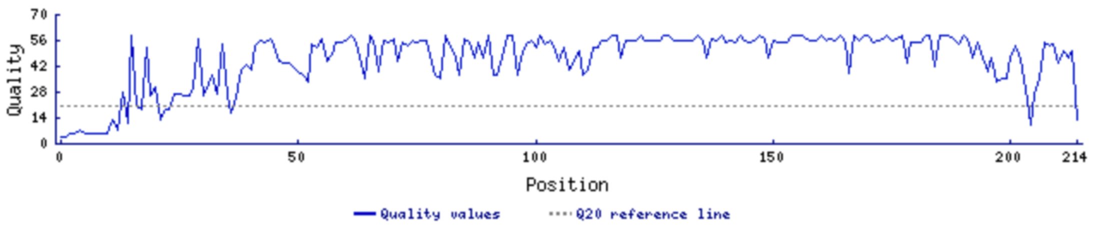

Read Length	215 b	Trimmed Length	206 b	Pos. 10-215
Q20	194 b	Q20/len	0.90	
Q40	155 b	Q40/len	0.72	

Sequence

Previous Revision	n/a	Current Revision	1	Next Revision	n/a	Latest Revision	1

Links: View Revision History

```
>3P_1RR_WPG1374-user_added CHROMAT_ID=1601335
gtcattgatggttgtggtttgttagtagtcgtgctcaaataacttcgtatagcatacatt     60
atacgaagttatagaaggaagaaattagattcagaaatctctctcttcccttctcccct    120
ctccttctttctctcctatctattgatacagggtaaaattggaaggtggggaataaagga    180
tgggaaaaagaagaaccactacaaagtggcaaaga
```

Targeted 3' loxP

Figure S3

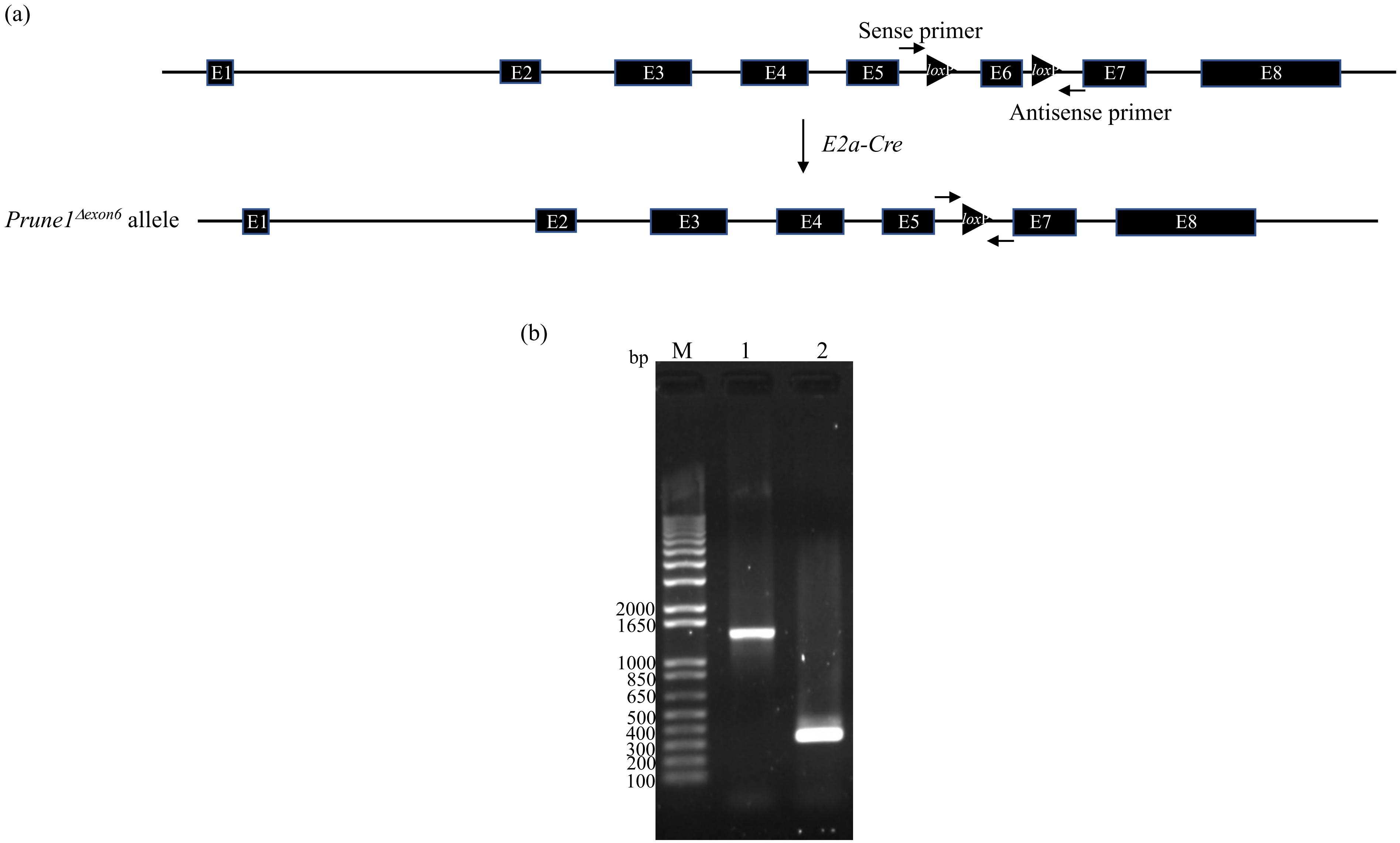

(a)
Sense primer
E1
E2
E3
E4
E5
loxP
E6
loxP
E7
E8
Antisense primer
E2a-Cre
Prune1Δexon6 allele
(b)
bp
M
1
2
2000
1650
1000
850
650
500
400
300
200
100

Figure S4
